# The Toil of Shop-Girls

**Published:** 1875-06

**Authors:** 


The Evil of Shop-Girls.—The custom of compelling shop-girls to stand up all the long day behind the counter, is selfish, cruel, and useless; especially selfish on the part of the proprietor, who requires the women to stand all the time, whether serving customers or not, merely that they may appear on the alert to wait on those who call. To stand from seven or eight in the morning till six, eight, or ten o'clock at night—as is the custom at certain stores—with a short time at mid-day for dinner, would weary any man. But to exact such service from girls and women! Their physical powers are, it is well known, much weaker than those of men; at any rate, and by their anatomical and physiological peculiarities, they are entirely unfit for bearing this specially severe toil, namely, standing all day long. Medical men who practice largely among women are constantly witnessing the terrible consequences of this most cruel "rule of the establishment."

